# Cortical Neuroplasticity and Cognitive Function in Early-Stage, Mild-Moderate Hearing Loss: Evidence of Neurocognitive Benefit From Hearing Aid Use

**DOI:** 10.3389/fnins.2020.00093

**Published:** 2020-02-18

**Authors:** Hannah Anneli Glick, Anu Sharma

**Affiliations:** Brain and Behavior Laboratory, Department of Speech, Language, and Hearing Science, Center for Neuroscience, Institute of Cognitive Science, University of Colorado Boulder, Boulder, CO, United States

**Keywords:** age-related hearing loss (ARHL), cortical visual evoked potentials (CVEPs), visual cross-modal re-organization, hearing aids, speech perception, cognition

## Abstract

Age-related hearing loss (ARHL) is associated with cognitive decline as well as structural and functional brain changes. However, the mechanisms underlying neurocognitive deficits in ARHL are poorly understood and it is unclear whether clinical treatment with hearing aids may modify neurocognitive outcomes. To address these topics, cortical visual evoked potentials (CVEPs), cognitive function, and speech perception abilities were measured in 28 adults with untreated, mild-moderate ARHL and 13 age-matched normal hearing (NH) controls. The group of adults with ARHL were then fit with bilateral hearing aids and re-evaluated after 6 months of amplification use. At baseline, the ARHL group exhibited more extensive recruitment of auditory, frontal, and pre-frontal cortices during a visual motion processing task, providing evidence of cross-modal re-organization and compensatory cortical neuroplasticity. Further, more extensive cross-modal recruitment of the right auditory cortex was associated with greater degree of hearing loss, poorer speech perception in noise, and worse cognitive function. Following clinical treatment with hearing aids, a reversal in cross-modal re-organization of auditory cortex by vision was observed in the ARHL group, coinciding with gains in speech perception and cognitive performance. Thus, beyond the known benefits of hearing aid use on communication, outcomes from this study provide evidence that clinical intervention with well-fit amplification may promote more typical cortical organization and functioning and provide cognitive benefit.

## Introduction

Age-related hearing loss (ARHL), or presbycusis, affects more than 30% of adults over age 50 years and its prevalence roughly doubles with each decade of life, making it the third leading chronic health condition among aging adults (Agrawal et al., 2008). Hearing aids and cochlear implants may restore audibility in ARHL, yet less than 15% of adults who could benefit from hearing aids in the United States use them (Chien and Lin, 2012) and this statistic is even lower (8%) among those adults who could benefit from cochlear implants (Holder et al., 2018). For the small percentage of adults who do seek treatment, treatment is sought out late, typically 7–10 years after initial hearing loss onset (Davis et al., 2007). Access and affordability issues likely complicates the hearing healthcare landscape for aging adults in the United States and there currently exist no best practice guidelines for screening and management of ARHL (Barnett et al., 2017).

Beyond the well-known negative effects of ARHL on communication, quality of life, physical functioning, and psychosocial status, ARHL has also been linked to cognitive decline. For example, large-scale epidemiological studies indicate a strong association between ARHL and risk for mild cognitive impairment and dementia, as well as accelerated decline in cognitive function over time (Lin, 2011; Lin et al., 2011; Thompson et al., 2017; Wei et al., 2017; Zheng et al., 2017; Ford et al., 2018; Loughrey et al., 2018). Though a lack of strong evidence on the long-term protective effects of clinical treatment of hearing loss on cognitive function exists, hearing loss is a potentially modifiable risk factor for cognitive decline (Livingston et al., 2017), warranting further investigation from a public health perspective (President’s Council of Advisors on Science and Technology [PCAST], 2015; National Academies of Sciences Engineering and Medicine [NASEM], 2016). One hypothesis explaining the hearing loss-dementia link is that decreased or degraded input to the auditory cortex makes listening more effortful, requiring greater top-down sensory, attentional, and cognitive compensation, which may in turn decrease available resources that can be contributed to other tasks, potentially negatively affecting downstream cognitive function (Pichora-Fuller and Singh, 2006; Schneider et al., 2010; Tun et al., 2012; Pichora-Fuller et al., 2016).

Cross-modal re-organization is a form of cortical compensation observed in deafness and lesser degrees of hearing loss, whereby the auditory cortex is recruited or “re-purposed” by intact visual and somatosensory modalities (Bavelier and Neville, 2002; Strelnikov et al., 2013; Glick and Sharma, 2017). For example, adults with mild-moderate ARHL exhibit more extensive recruitment of auditory cortex during visual motion and face processing tasks relative to NH subjects (Campbell and Sharma, 2014; Stropahl and Debener, 2017). Similarly, vibrotactile stimulation in adults with ARHL elicits more extensive cross-modal neural activity in the auditory cortex (Cardon and Sharma, 2018). Both visual and somatosensory cross-modal re-organization are associated with poorer auditory speech perception outcomes (Campbell and Sharma, 2014; Cardon and Sharma, 2018), but the extent to which these neuroplastic changes influence cognitive outcomes has not been investigated.

In this study, we used high-density electroencephalography (EEG) to record visual evoked potentials (CVEPs) in response to visual stimuli in a group of adults with mild-moderate ARHL and in age-matched normal hearing (NH) controls to assess the relationship between visual cortical neuroplasticity, speech perception and cognitive function. We then fit the group of adults with ARHL with bilateral hearing aids to examine how increased audibility from amplification influenced cortical neuroplasticity, speech perception, and cognitive outcomes.

## Materials and Methods

### Ethics Approval Statement

This study was carried out in accordance with the recommendations of Belmont Report. The protocol was approved by the Institutional Review Board at the University of Colorado Boulder. All subjects provided written informed consent prior to participation in the study in accordance with the Declaration of Helsinki.

### Subjects

A total of 41 adults took part in this study (mean age = 64 years, *SD* = 4.68). Subjects were native speakers of English, with no reported neurological impairment and reported normal or corrected-to-normal visual acuity. Thirteen adults comprised the NH control group (mean age = 62.62 years, *SD* = 4.91) and 28 adults comprised the ARHL experimental group (mean age = 65.4 years, *SD* = 4.23). Independent samples *t*-tests were conducted to confirm that groups did not significantly differ in terms of age [*t*(39) = 1.621, *p* = 0.980] or gender [*t*(39) = 0.394, *p* = 0.356]. It should be noted that it was difficult to recruit subjects in this age-range with normal hearing, likely due to high prevalence of ARHL. None of the ARHL subjects reported hearing aid use prior enrollment in the study.

There was no difference between groups on a variety of known demographic risk factors for hearing loss including smoking [*t*(39) = 1.508, *p* = 0.140], noise exposure [*t*(39) = 1.643, *p* = 0.109], or hypertension [*t*(39) = −0.116, *p* = 0.908]. No subjects reported history of diabetes or clinical depression. The two groups did not differ in terms of education level [*t*(39) = −0.975, *p* = 0.335] or handedness [*t*(40) = 1.030, *p* = 0.309]. As expected with the presence of hearing loss, report of tinnitus was significantly higher in the ARHL group [*t*(39) = 4.210, *p* < 0.001], with 68% ARHL subjects reporting some level of tinnitus. Interestingly, self-report of balance problems was significantly higher in the hearing loss group [*t*(39) = 2.030, *p* = 0.049], with 25% of hearing loss subjects reporting balance disturbances and/or falls in the past year.

### Inclusion Criteria

**FIGURE 1:**
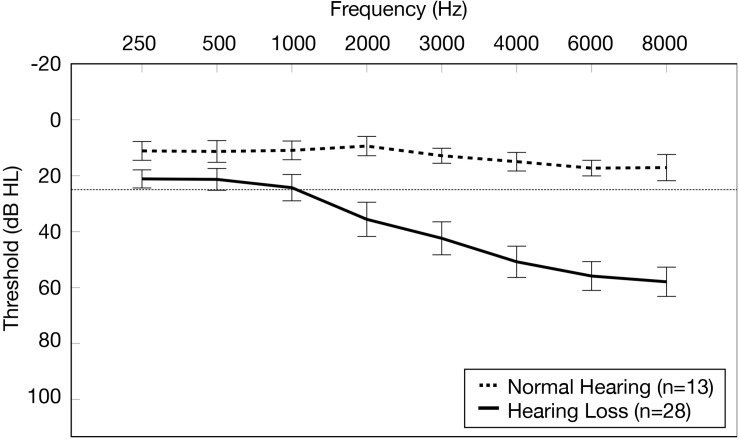
Average pure tone air conduction thresholds for the normal hearing group and age-related hearing loss group. Average pure tone air conduction thresholds across the two ears (0.25–8.0 kHz) are displayed for the normal hearing group (*n* = 13) (dashed line) and the group with mild-moderate age-related hearing loss (*n* = 28) (solid line). Frequency (Hz) is displayed on the horizontal axis and pure tone air conduction thresholds in decibels hearing level (dB HL) are displayed on the vertical axis. The dotted line on the y-axis indicates the clinical cutoff for normal hearing thresholds (25 dB HL). The bars display 95% confidence intervals at each threshold for each group.

Audiological inclusion criteria for the NH group were defined as pure tone audiometric behavioral thresholds for both ears ≤ 25 dB HL from 0.25 to 8.0 kHz, no presence of an air-bone gap (≥15 dB HL at 2 or more adjacent frequencies), and no sign of interaural asymmetry (≥15 dB HL at 2 or more frequencies). Audiological inclusion criteria for the ARHL group was defined as a high frequency pure tone average (HFPTA) (2, 4, 6kHz) > 25 dB HL in both ears, no presence of an air-bone gap (≥15 dB HL at 2 or more adjacent frequencies), and no sign of interaural asymmetry (≥15 dB HL at two or more frequencies). Because pure tone average (PTA) thresholds (0.5, 1, 2 kHz) [*t*(39) = −2.44, *p* = 0.81] and high frequency pure tone average (HFPTA) thresholds (2, 4, 6 kHz) [*t*(39) = −1.52, *p* = 0.137] between the right and left ears were not statistically different among subjects, averaged audiometric thresholds across the 2 ears were computed and used for subsequent analyses for each group. Average pure tone air conduction thresholds for each group and corresponding 95% confidence intervals are depicted in Figure 1. Average PTA thresholds were 16.5 dB HL poorer in the ARHL group (average = 27.08 dB HL, *SD* = 10.41) compared to the NH group (average = 10.58 dB HL, *SD* = 5.23) [*t*(39) = 5.386, *p* < 0.001]. Average HFPTA thresholds were approximately 33.5 dB HL poorer in the ARHL group (average = 47.44 dB HL, *SD* = 11.54) compared to the NH group (average = 13.91 dB HL, *SD* = 3.77) [*t*(39) = 10.17, *p* < 0.001]. On average, the ARHL group demonstrated a mild sloping to moderate hearing loss and the NH group demonstrated clinically normal hearing thresholds.

Subjects in the ARHL group were required to wear their hearing aids at least 5 h/day for inclusion in 6 months follow-up analyses. Of the 28 ARHL subjects initially enrolled in the study at baseline, a total of 21 subjects (average age = 64.38 years, *SD* = 4.03) met this criterion. The remaining 7 ARHL subjects were removed from 6 months follow-up analyses due to inability to adjust to hearing aids and/or insufficient hearing aid use (*n* = 5) (occurring in the first 2 weeks to 3 months after enrollment in the study) or inability to return for 6 months follow-up testing (*n* = 2).

### Hearing Aid Fitting and Verification

Prior to baseline testing, the ARHL group was acutely fit with bilateral receiver-in-the-ear hearing aids from a single manufacturer. The purpose of acute hearing aid fitting was to negate potential confounding effects of audibility on test performance at the baseline evaluation. Hearing aids were programmed in the manufacturer fitting software. Appropriate receiver size (60-power receiver for thresholds < 60 dB HL 0.25–8.0 kHz; 85-power receiver for thresholds ≥ 60 dB HL 0.25–8.0 kHz) and appropriate non-custom acoustic coupling options (open dome, vented, or closed domes) were selected for each ARHL subject based on the degree of hearing loss. Settings for noise reduction, microphone mode, noise management, and binaural broadband controls were set to manufacturer defaults. Acoustic feedback reduction algorithms were not applied due to the potential for these algorithms to affect ideal frequency-gain characteristics and to promote generalizability across manufacturers since these algorithms vary between manufacturers. Instead, if significant feedback was present, modifications to the acoustic coupling (e.g., selecting a more occlusive dome) were made to prevent acoustic feedback.

Probe-microphone measures were performed to verify hearing aid fittings for the ARHL subjects using the Audioscan probe-microphone verification system. Hearing aid gains were adjusted to meet NAL-NL2 prescribed targets between 0.25 and 4.0 kHz for soft (55 dB SPL), medium (65 dB SPL) and loud (75 dB SPL) speech inputs. Maximum Power Output (MPO) was also measured with a swept tone stimulus to approximate uncomfortable loudness levels (UCL). Probe microphone measurements were ± 5 dB of NAL-NL2 targets from 0.25 to 4.0 kHz for all ARHL subjects, indicating adequate audibility. The average difference between actual and prescriptive gain for the 65 dB SPL input was 1.76 above NAL-NL2 targets for the right ear (*SD* = 2.58) and + 0.96 dB above NAL-NL2 targets for the left ear (*SD* = 3.16) from 0.25 to 4.0 kHz.

### Hearing Aid Follow-Up and Data Logging

The ARHL group returned for routine hearing aid maintenance checks and data logging approximately 2 weeks, 1, 3, and 6 months post-treatment in order to ensure hearing aids were functioning properly and to document average daily hearing aid use using the manufacturer fitting software. At the final 6 months follow-up visit, cumulative usage time over each visit computed for each subject. Only those ARHL subjects who wore their hearing aids for minimum of 5 h/day (*n* = 21) were included in final 6 months follow-up analyses. Average hearing aid use in these subjects ranged between 5.10 and 14.02 h/day (mean = 9.84 h/day, *SD* = 2.96).

### Cortical Visual Cortical Evoked Potential Testing

Cortical visual evoked potentials (CVEPs) were measured for NH and ARHL subjects in an unaided condition using 128-channel high-density EEG (GSN-Hydrocel 128, Electrical Geodesics, Inc.). CVEPs were recorded using NetStation 5 software (Electrical Geodesics, Inc.) at a sampling rate of 1000 Hz with a band-pass filter set at 0.1–200 Hz. Subjects were seated in an electro-magnetically shielded sound booth and CVEP responses were elicited via a visual motion stimulus (radially modulated grating or star-circle pattern), providing the percept of apparent motion. The visual stimulus was adapted from Doucet et al. (2006) and used in several previous studies in our laboratory (Campbell and Sharma, 2014, 2016; Sharma et al., 2016). Three hundred trials were presented (150 star, 150 circle stimulus presentations) at an inter-stimulus interval of 495 ms and pre-stimulus interval of 100 ms (595 ms recording window). Subjects were instructed to focus on the black dot in the center of the pattern without shifting their gaze. Stimuli were presented via E-Prime 2.0 stimulus presentation software and displayed on a flat screen LCD television at a viewing distance of approximately 42 inches.

### Cortical Visual Evoked Potential Waveform Analysis and Current Density Source Reconstruction

CVEP data for each subject were pre-processed offline by applying a high-pass filter (1 Hz). Continuous data were segmented around the stimulus presentation recording window and data were exported from NetStation 5 to Matlab^TM^ (The MathWorks^®^, Inc.) via EEGLab (Delorme and Makeig, 2004), where baseline correction (to the 100 ms pre-stimulus recording window), bad channel rejection (± 100 μV), bad epoch rejection, re-referencing (to the common average reference), and down sampling (from 1 to 0.25 kHz, to reduce processing time) were performed. Average CVEP responses for each subject were then computed by averaging CVEP responses across several electrodes corresponding to cortical regions of interest (ROIs) on the scalp: Occipital (E71, E76, E70, E75, E83, E74, E82), right temporal (E110, E104, E109, E103, E93, E86, E98, E102, E108), and left temporal (E35, E40, E41, E36, E45, E46, E47, E42, E52) ROIs. ROIs were selected *a priori* based upon previous studies where differences in cortical activation patterns were observed in adults with mild-moderate ARHL using this same stimulus (Campbell and Sharma, 2014) and evidence from previous neuroimaging studies (PET, fMRI, intracranial CVEP recordings) in typical subjects (Dupont et al., 2003; Bertrand et al., 2012; Kellermann et al., 2012) using the same or similar visual motion stimuli. After computing average CVEP responses for each subject across each ROI, peak latencies and amplitudes were extracted for statistical analyses. Peak latency and peak amplitudes were defined at the midpoint of the peak for each CVEP waveform component (P1, N1, P2). Individual waveforms were averaged together to create a grand-averaged waveform for each group (NH and ARHL) at baseline and for the ARHL at 6 months follow-up.

Group cortical source localization analyses were then performed on CVEP data. An independent components analysis (ICA) was applied to pre-processed CVEP data for each subject to identify spatially fixed and temporally independent components underlying each component (P1, N1, P2) in the CVEP response according to the timeframe in which the component occurred (Makeig et al., 1997; Delorme et al., 2012). ICA components accounting for the greatest percent variance for each of the CVEP component were kept, while remaining ICA components were regarded as artifact/noise and discarded. The ICA-pruned CVEP data for individual subjects were then exported from Matlab^TM^ into Curry7^TM^ Neuroimaging Suite (Compumedics Neuroscan^TM^), where cortical source modeling was performed. Here, grand average ICA-pruned CVEP waveforms for the NH group at baseline and the ARHL group at baseline and 6 months follow-up visits were computed. Current density source reconstruction (CDR) was performed to visualize group and treatment differences in cortical activation patterns. To achieve this, a second ICA was performed on the grand averaged data for each group to identify components with the highest SNR. A head model was then created and standardized using the boundary element method (BEM) (Fuchs et al., 2002). Next, CDRs were computed via standardized low-resolution electromagnetic tomography (sLORETA). sLORETA is a statistical method that estimates current densities with low localization error (Pascaul-Marqui, 2002; Grech et al., 2008). The resultants CDRs were projected onto an average adult structural MRI (provided by the Montreal Neurological Institute). CDRs are depicted by a graded color scale (F-statistic) indicating the statistical likelihood of cortical activity in each region. This described protocol has been used in our laboratory to observe changes in visual cross-modal plasticity in adults and children with hearing loss at the single-subject and group level (Campbell and Sharma, 2014, 2016; Sharma et al., 2015, 2016; Cardon and Sharma, 2018).

### Speech Perception in Noise Testing

Auditory speech perception in noise was measured in an unaided condition for NH group and ARHL groups at baseline and in an aided condition in the ARHL group at 6 months follow-up visit using the QuickSIN^TM^ test. The QuickSIN^TM^ is a standardized assessment of sentence-level auditory speech perception in background noise (Etymotic Research, 2001; Killion et al., 2004). Two randomly selected recorded lists of 6 sentences (5 key words per sentence) were presented in the context of 4-talker babble noise. Stimuli were presented in a binaural condition via a speaker located at 0° azimuth at a level of 60 dB SPL (conversational speech level). The sentences in each list varied in signal-to-noise ratio (SNR), beginning at 25 dB SNR (easiest) for the first sentence and decreased in 5 dB steps with each subsequent sentence (most difficult). The test is scored in terms of the dB SNR loss, or the dB SNR required for the subject to score 50% of the words correct (threshold), relative to NH adult listeners, with a lower score indicating better auditory speech perception in noise performance and a higher score indicating poorer auditory speech perception in noise performance.

The Arizona Auditory-Visual (AzAv) test was administered for assessment of auditory-visual speech perception in noise (Dorman et al., 2016). The test was administered in an unaided condition at baseline for NH group and in an aided condition at baseline and 6 months follow-up visits for the ARHL group in order to negate potential confounding effects of audibility on test performance. The AzAv was adapted from sentence materials in Macleod and Summerfield (1987, 1990) and developed using methodology of Spahr et al. (2012) in creation of the AzBio, a routinely used auditory-only clinical assessment of speech perception in background noise. The AzAv has been validated in NH and cochlear implanted adults in a series of previous studies reported in Dorman et al. (2016). The test contains 10 lists, with each list comprised of 15 sentences (3 key words per sentence). Sentences spoken by a target talker are presented in the context of multi-talker babble. The test was administered in a binaural condition via a speaker located 0° azimuth, with target sentences presented at a level of 60 dB SPL (conversational speech level). Visual (lip-reading) stimuli were presented on an LCD television at a viewing distance of approximately 42 inches. Several practice lists were first administered in an auditory-only condition, varying the SNR in 2 dB increments (starting at the SNR determined by the QuickSIN^TM^ test) to determine the level at which the subject repeats approximately 40–50% of words correct (to prevent ceiling effects). Next, 2 randomly selected lists were presented in an auditory-only condition and 2 randomly selected lists were presented in an auditory-visual condition. Performance on the AzAv test is scored in terms of visual (lip-reading) benefit, by subtracting average performance (in percent key words correct) in the auditory-only condition from the auditory-visual condition, providing a percent benefit score from the addition of visual (lip-reading) cues.

### Cognitive Testing

Cognitive tests were administered in an unaided condition for the NH group and in an aided condition for the ARHL group at baseline and 6 months follow-up visits to negate potential confounding effects of audibility on test performance for the ARHL group. Testing was conducted in a quiet room for all participants to prevent negative effects of noise on test performance for all subjects (Dupuis et al., 2015). The cognitive measures selected probe several cognitive sub-domains: Global cognitive function (Montreal Cognitive Assessment – MoCA) (Nasreddine et al., 2005), executive function (Behavioral Dyscontrol Scale II – BDS-2) (Grigsby and Kaye, 1996), processing speed (Symbol Digits Modalities Test – SDMT) (Smith, 1982), visual working memory (Reading Span Test – RST) (Daneman and Carpenter, 1980; Rönnberg et al., 1989), and auditory working memory (Word Auditory Recognition and Recall Measure) (WARRM) (Smith et al., 2016). The aforementioned sub-domains and associated neuropsychological tools were selected based on theoretical predictions about which sub-domains would be most affected by ARHL and previous investigations where impairments were observed in ARHL subjects (Lin, 2011; Lin et al., 2011, 2013; Loughrey et al., 2018). Test-retest reliability over repeated testing of each cognitive measure is described in the Discussion section.

### Subjective Hearing Aid Outcome Measures

To validate hearing aid outcomes in the ARHL group at the 6 months follow-up visit, the Client Oriented Scale of Improvement (COSI) (Dillon et al., 1997), the International Outcome Inventory for Hearing Aids (IOI-HA) (Cox et al., 2002, 2003), and the Satisfaction with Amplification in Daily Living (SADL) scales (Cox et al., 2003) were administered. These questionnaires are routinely used in the clinical setting and provide valuable information regarding self-perceived benefit and satisfaction with hearing aids. The COSI measure asks hearing loss subjects to identify and rank in order up to 5 specific listening situations where they hope to see improvements with hearing aids before hearing aid fitting. Subjects then rate the degree of change in hearing ability on 5-point scale (1 = worse, 2 = no difference, 3 = slightly better, 4 = better, and 5 = much better) and their final hearing ability on a 5-point scale (1 = hardly ever, 2 = occasionally, 3 = half the time, 4 = most of the time, and 5 = almost always) in each of these self-identified listening situation after hearing aid fitting. An averaged degree of change score and final ability score is computed across these listening situations (Dillon et al., 1999). While the COSI is not a standardized measure, it probes situations perceived to be most important to each individual. The IOI-HA is a standardized 7-item survey that targets several different outcome domains: Daily use, benefit, residual activity limitations, satisfaction, residual participation restrictions, impact on others, and quality of life. ARHL subjects were asked to provide a rating for each item on a 5-point scale (1 = severe, 2 = moderately-severe, 3 = moderate, 4 = mild, 5 = none), where a lower score indicates poorer outcome and a higher score indicates higher outcome for each item (Cox and Alexander, 2002; Cox et al., 2002, 2003; Kramer et al., 2002; Noble, 2002; Stephens, 2002). The SADL is a standardized 15-item survey targeting elements most important to patient satisfaction. Subjects are asked to indicate the relative importance each item on a 7-point scale. The questionnaire was administered at the 6 months post-treatment assessment visit. The questionnaire yields a global satisfaction score as several sub-scores across the following domains: Positive effects, service, negative features, and personal image (Cox and Alexander, 1999, 2001). An average score was calculated for each sub-score category by summing ratings for each item in that category and dividing by the total number of items in that category. A global score was also computed by averaging ratings across all items and dividing by the total number of items.

### Statistical Analysis

Statistical analyses were conducted using the Statistical Package for Social Sciences (SPSS) version 25. Histograms, Q-Q plots, and significance tests (Shapiro–Wilk test, Levene test) were first computed to assess potential violation in assumptions of normality and homogeneity of variance for all variables. Visual inspection and outlier analyses were also performed.

Two-tailed independent sample *t*-tests were used to assess differences in the cortical, speech perception, and cognitive outcome variables between NH group and ARHL group at baseline. A series of two-tailed, paired samples sample *t*-tests were applied to assess pre-post treatment effects with hearing aids on cortical, speech perception, and cognitive variables in the ARHL group at the 6 months follow-up visit. Because multiple comparisons were made to assess CVEP (P1, N1, P2) latencies across the different 3 ROIs, a Bonferroni correction was applied (alpha error divided by number of tests) to reduce chance of Type I error, reducing the alpha level from α = 0.05 to α = 0.017. The same correction was applied for assessing CVEP (P1, N1, P2) amplitudes across the 3 ROIs.

To assess the association between CVEP latencies, speech perception, cognitive performance, and degree of hearing loss within the group of adults with ARHL at baseline and at 6 months follow-up, Pearson’s correlation coefficients were computed. Because comparisons were made between the 3 different CVEP components and cognitive outcome measures, a Bonferroni correction was applied during these analyses to reduce chance of Type I error, reducing the alpha level from α = 0.05 to α = 0.017.

## Results

### Group Differences in Cortical Visual Evoked Potential Latencies and Amplitudes at Baseline

**FIGURE 2:**
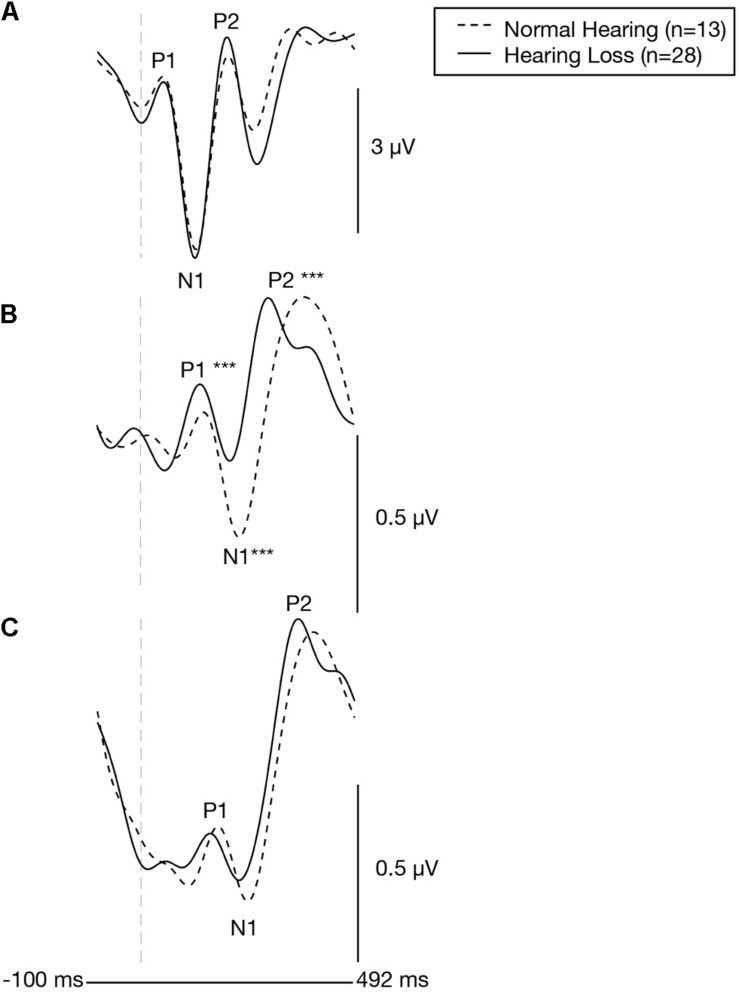
Baseline group differences in cortical visual evoked potentials across an occipital, right temporal, and left temporal region of interest. Grand-averaged CVEP waveforms for the normal hearing group (*n* = 13) and the age-matched group of adults with early-stage, age-related hearing loss (*n* = 28) are depicted for the occipital region of interest **(A)**, the right temporal region of interest **(B)**, and left temporal region of interest **(C)**. Time (milliseconds) is displayed on the horizontal axis and amplitude (μV) is displayed on the vertical axis. Asterisks show level of significance (***p ≤ 0.001) for differences in CVEP latencies between the two groups. The hearing loss group showed significantly earlier CVEP P1, N1, and P2 latencies over the right temporal region compared to the normal hearing group.

Plots of the grand average CVEP waveforms for the NH and ARHL groups across the occipital, right temporal, and left temporal ROIs are depicted in Figure 2. CVEP responses in the NH and ARHL groups are marked by the presence of all 3 obligatory P1, N1, and P3 CVEP components. Morphological patterns are similar to the findings reported in Campbell and Sharma (2014) using the same stimulus in a smaller group subjects with NH and mild-moderate ARHL. Independent samples *t*-tests indicated no significant differences in P1, N1, or P2 peak latencies or amplitudes between the NH and ARHL in the occipital or left temporal ROI. However, significant differences in P1, N1 and P2 peak latencies were observed in the right temporal ROI (α < 0.0055 level). Relative to the NH group, the ARHL group exhibited significantly earlier P1 [*t*(39) = −4.65, *p* < 0.001], N1 [*t*(39) = −5.36, *p* < 0.001], and P2 CVEP latencies [*t*(39) = −3.42, *p* = 0.001] in the right temporal ROI (Table 1). Large effect sizes (Cohen’s *d*-values) were observed for the P1 (*d* = 1.66), N1 (*d* = 1.82), and P2 (*d* = 1.21) components.

**TABLE 1:** Baseline cortical visual evoked potential latencies over a right temporal region of interest.

**Component**	**Average latency (ms)**		**Standard deviation**		**95% confidence interval**		**Statistic *t*(39) (*p*-value)**	**Effect size (Cohen’s d)**
					
	**NH**	**ARHL**	**NH**	**ARHL**	**NH**	**ARHL**		
P1	128	99	15.62	19.1	118–137	92–106	−4.65, (< 0.001)	1.66
N1	175	134	20.38	24.42	163–187	124–143	−5.36, (< 0.001)	1.82
P2	242	203	24.62	38.42	228–258	188–218	−3.42, (0.001)	1.21

**Average peak latencies, standard deviations, 95% confidence intervals, statistical significance values, and effect size are provided for the age-related hearing loss (HL) (n = 28) group and the normal hearing (NH) group (n = 13) in a right temporal region of interest. Significantly earlier P1, N1, and P2 latencies are observed in the HL group.**

### Group Differences in Cortical Visual Evoked Potential Current Density Source Reconstruction Patterns at Baseline

**FIGURE 3:**
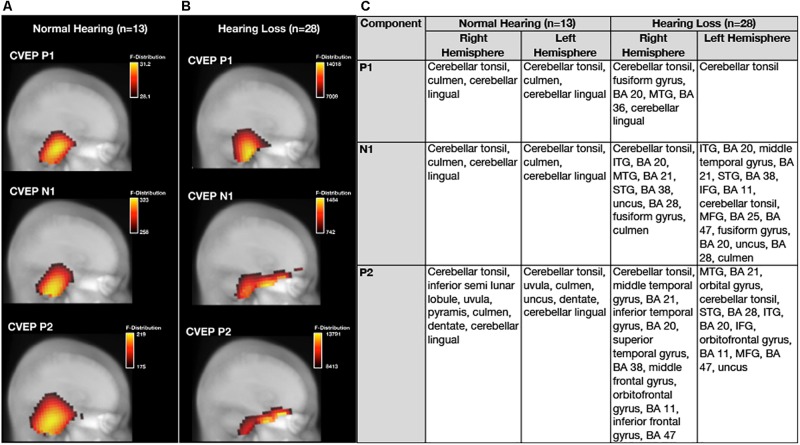
Baseline group differences in cortical source activation patterns elicited by visual motion stimuli. Baseline Current density source reconstructions (CDR) for the P1, N1, and P2 cortical visual evoked potential components for the normal hearing group (*n* = 13) are depicted in **(A)** and CDRs for the hearing loss group (*n* = 28) are depicted in **(B)**. 3D current density source reconstructions obtained via standardized low-resolution brain electromagnetic tomography (sLORETA) are projected on a 2D depth-buffered structural magnetic resonance image (Maximum Intensity Projection), providing visualization of the voxels with the highest likelihood of activation. The color scale to the right of each figure shows the statistical likelihood of activation (F-statistic), from lowest (red) to highest (yellow) probable current density. **(C)** Describes regions of cortical source activity for each CVEP component, including Brodmann areas (BA), for the normal hearing and age-related hearing loss group by rank in order highest to lowest likelihood of activation (F-statistic).

Average baseline CDRs for the NH and untreated ARHL groups are depicted for each CVEP component (P1, N1, and P2) in Figure 3. 3D CDRs are displayed on a Maximum Intensity Projection (MIP) (a 2D depth-buffered MRI), providing visualization of the voxels with the highest likelihood of activation. The gradient color scale to the right of each figure indicates the statistical likelihood of activation (F-statistic), from lowest (red) to highest (yellow) probable current density computed via sLORETA. Table 1 lists the cortical regions of activity for each component in the CVEP response in order of highest to lowest likelihood of activation.

As can be observed in Figure 3, the visual motion stimulus elicited activity in bilateral occipital and cerebellar cortical regions for all CVEP components in the NH group. These cortical sources are similar to those reported in fMRI and PET studies using similar visual motion stimuli to ours (Dupont et al., 2003; Kellermann et al., 2012) and a previous intracranial CVEP study using the same visual motion stimulus as ours (Bertrand et al., 2012). In the ARHL group, the visual motion stimulus elicited activation over bilateral occipital and cerebellar regions for the P1 CVEP component. For the N1 and P2 component, occipital and cerebellar cortical activation was observed in addition to activation of regions of the auditory cortex (e.g., superior, middle, and inferior temporal gyrus), evidence of visual cross-modal re-organization in the mild-moderate ARHL group. Evidence of cross-modal re-organization as evidenced by activation of regions of auditory cortex to the same visual motion stimulus has been previously reported by Campbell and Sharma (2014) in a group of adults with mild-moderate hearing loss. Further, in addition to evidence of cross-modal recruitment of auditory cortex in the ARHL group at baseline, the ARHL group also exhibited pre-frontal and frontal cortex (orbital gyrus, inferior frontal gyrus, and middle frontal gyrus, predominately in the left hemisphere) activity for the later N1 and P2 CVEP components at the baseline evaluation.

### Group Differences in Speech Perception in Noise at Baseline

**FIGURE 4:**
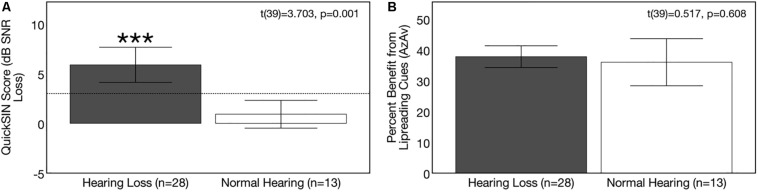
Baseline group differences in speech perception in noise. Average scores on speech perception measures in the age-related hearing loss group (*n* = 28) are depicted in gray and average scores for the normal hearing group (*n* = 13) are depicted in white. Black bars indicate 95% confidence intervals for each group. Asterisks indicate level of significance (***p ≤ 0.05). **(A)** Depicts average binaural auditory speech perception in noise scores (QuickSIN^TM^). Scores are plotted in terms of the dB signal-to-noise ratio loss (dB SNR), representing the dB SNR required for the participant to score 50% of words in a sentence correct relative to normal hearing listeners. A higher score indicates poorer auditory speech perception in noise performance. The dotted line indicates the cutoff score (3 dB SNR) for normal function in background noise. The hearing loss group performed significantly poorer on the QuickSIN^TM^ test. **(B)** Depicts average benefit from visual (lipreading) cues on a binaural auditory-visual speech perception in noise test (AzAv). Scores indicate the percent difference score in an auditory-only condition relative to an auditory-visual condition, where a higher score indicates greater benefit from the addition of visual (lip-reading) cues. No significant difference was observed between groups in terms of lip-reading benefit.

Average baseline auditory speech perception scores and corresponding 95% confidence intervals for the NH and ARHL groups are depicted in Figure 4A. QuickSIN^TM^ scores were significantly poorer in the hearing loss group compared to the NH group at baseline [*t*(39) = 3.703, *p* = 0.001]. Scores indicated a mild deficit in background noise (3–7 dB SNR) in the ARHL group (average = 5.89 dB SNR loss, *SD* = 4.55) and normal performance (0–3 dB SNR) in the NH group (average = 0.92 dB SNR loss, *SD* = 2.31) (Killion et al., 2004). Average speech perception in noise scores on this test in the hearing loss group are comparable to results in adults with similar degree of sensorineural hearing loss reported in previous studies (Killion et al., 2004; Wilson et al., 2007) and are consistent with the mild-moderate range of hearing loss in our ARHL study sample.

Average baseline visual (lip-reading) benefit scores on the auditory-visual speech perception in noise scores for the NH and ARHL groups are shown in Figure 4B. Average benefit from visual cues on the AzAv test across the NH and ARHL groups was 37.21% (*SD* = 10.24) and there was no significant difference in performance between the NH and ARHL groups [*t*(39) = 0.517, *p* = 0.608], indicating that adults with early-stage (mild-moderate) hearing loss do not derive greater relative benefit from visual (lip-reading) cues compared to age-matched NH control subjects. This finding is comparable to previously reported visual benefit using the same AzAv test materials in cochlear implant recipients, where average benefit from visual cues was 32–44% (Dorman et al., 2016) in studies of older adult listeners using similar auditory-visual speech perception measures (Cienkowski and Carney, 2002; Sommers et al., 2005). The relative benefit from visual (lip-reading) cues described in our study is also comparable to benefit described in younger adult populations under acoustically degraded listening situations (Sumby and Pollack, 1954; Grant and Seitz, 2000; Schwartz et al., 2004; Ross et al., 2007).

### Group Differences in Cognitive Function at Baseline

**FIGURE 5:**
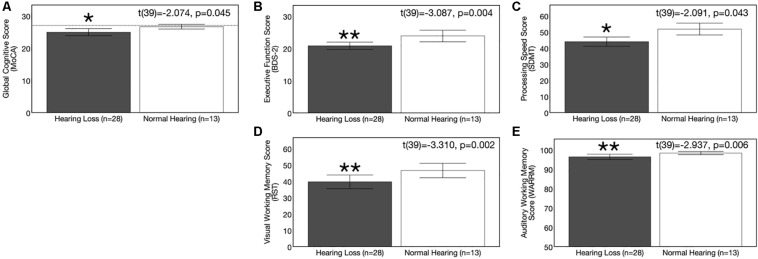
Baseline group differences in cognitive function. Average scores on cognitive measures in the age-related hearing loss group (*n* = 28) are depicted in gray and average scores for the normal hearing group (*n* = 13) are depicted in white. Black bars indicate 95% confidence intervals for each group. Asterisks indicate level of significance (**p ≤ 0.01, *p ≤ 0.05). **(A)** Depicts average global cognitive function on a screening measure (MoCA) for mild cognitive impairment. Higher scores in indicate higher global cognitive function, out of a total score of 30 points. The dotted reference line on the y-axis indicates the cutoff score (≤ 27) indicating risk for mild cognitive impairment. **(B)** Shows average executive function score (BDS-2). Higher scores in indicate better executive functioning, out of a total score of 27 points. **(C)** Depicts average processing speed score (SDMT). Higher scores in indicate faster processing speeds in a timed, 90 s digit-symbol matching task. **(D)** Shows average visual working memory score (RST) in percent words correctly recalled. Higher scores indicate higher visual working memory performance in a dual-task paradigm. **(E)** Displays average auditory working memory score (WARRM) in percent words correctly recalled. Higher scores indicate higher auditory working memory performance in a dual-task paradigm. The hearing loss group performed more poorly than the normal hearing group across all cognitive outcome measures assessed. Note: Cognitive testing in the hearing loss group was administered in an acutely aided condition to negate confounding effects of audibility on cognitive performance.

Average results on cognitive measures and corresponding 95% confidence intervals are depicted in Figures 5A–E. At baseline, the ARHL group performed significantly poorer than the NH group across all cognitive sub-domains: Global cognitive function, executive function, processing speed, visual working memory, and auditory working memory. Average global cognitive score (MoCA) was 1.69 points lower in the ARHL group (mean score = 24.93, *SD* = 2.80) compared to the NH group (mean score = 26.62, *SD* = 1.193) and this difference was statistically significant [*t*(39) = −2.074, *p* = 0.045]. Executive function scores (BDS-2) were 3.06 points lower in the ARHL group (mean score = 20.79, *SD* = 2.80) compared to the NH group, and this difference was also statistically significant [*t*(39) = −3.087, *p* = 0.004]. The ARHL group (mean score = 43.96, *SD* = 7.42) performed 7.81 points poorer on the processing speed measure (SDMT) compared to the NH group at baseline, and this difference was significantly significant (average score = 51.77, SD = 6.06) [*t*(39) = −3.310, *p* = 0.002]. Percent recall scores on the visual working memory task (RST) were 6.92% poorer in the hearing loss group (average recall score = 39.61%, *SD* = 10.81) compared to the NH group (average recall score = 46.53%, *SD* = 7.25) [*t*(39) = −2.091, *p* = 0.043]. Percent recall scores on the auditory working memory task (WARRM) were 11.39% poorer in the ARHL group (average recall score = 71.52%, *SD* = 13.36) compared to the NH group (average recall score = 82.01%, *SD* = 5.69) [*t*(39) = −2.937, *p* = 0.006, α < 0.01]. Together, these results suggest a negative impact on cognitive function even in mild hearing loss.

### Correlation Between Cortical Visual Evoked Potential Latencies and Behavioral Measures in Untreated, Age-Related Hearing Loss at Baseline

To evaluate the association between visual cortical cross-modal re-organization and behavioral outcomes, we correlated baseline CVEP latencies in ARHL group over the right temporal ROI to auditory performance (degree of hearing loss and auditory speech perception in noise), functional dependence on visual cues, and cognitive function.

**FIGURE 6:**
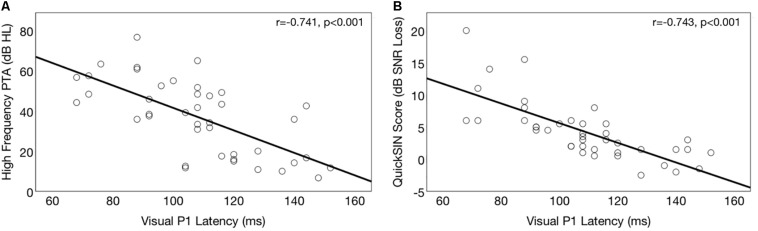
Association between baseline cortical visual evoked potential latencies (CVEP) and auditory function in the hearing loss group. **(A)** Depicts correlations between right temporal CVEP P1 peak latency (in milliseconds) and high frequency pure tone average (averaged across the right and left ear) in the ARHL group. A higher pure tone average (measured in dB hearing level, or dB HL) indicates more severe hearing loss. Earlier CVEP latency, considered an index of visual cross-modal re-organization, is associated with more extensive auditory deprivation in the high frequencies. **(B)** Depicts correlations between right temporal CVEP P1 peak latency (in milliseconds) and binaural auditory speech perception in noise (QuickSIN^TM^) performance for the ARHL group at baseline. A higher QuickSIN^TM^ score indicates poorer performance in background noise. Earlier CVEP latency, considered an index of visual cross-modal re-organization, is associated with poorer auditory performance in background noise.

Correlations between P1 CVEP and degree of hearing loss for the ARHL group are depicted in Figure 6A. A significant negative correlation was observed between HFPTA and P1 (*r* = −0.672, *p* < 0.001), N1 (*r* = −0.741, *p* < 0.001), and P2 (*r* = −0.572, *p* < 0.001) CVEP latencies in the right temporal ROI in the ARHL group at baseline, suggesting that more extensive cross-modal re-organization is apparent in greater degrees of hearing loss. This result is consistent with findings from Stropahl and Debener (2017) where degree of hearing loss and strength of visual cross-modal re-organization in the auditory cortex to visual stimuli were significantly associated in a group of adults with mild-moderate ARHL.

Correlations between CVEP P1 latency and auditory speech perception is shown in Figure 6B. A significant negative correlation was observed between auditory speech perception in noise on the and right temporal ROI CVEP peak latencies for the P1 (*r* = −743, *p* < 0.001), N1 (*r* = −0.643, *p* < 0.001), and P2 (*r* = −0.532, *p* < 0.001) in the untreated ARHL group. This finding suggests that earlier CVEP latencies, considered an index of more extensive visual cross-modal re-organization of auditory cortex, are associated with poorer auditory speech perception performance. This finding is consistent with previous studies in deaf adults (Doucet et al., 2006; Buckley and Tobey, 2011; Sandmann et al., 2012; Strelnikov et al., 2013; Chen et al., 2016) deaf children (Lee et al., 2001; Campbell and Sharma, 2016), and adults with mild-moderate hearing loss (Campbell and Sharma, 2014).

No significant was observed between CVEP latencies and dependence on visual (facial) cues in the ARHL group at baseline for any of the CVEP components [P1 (*r* = 0.070, *p* = 0.724), N1 (*r* = −0.123, *p* = 0.532), P2 (*r* = −0.41, *p* = 0.837)]. While a significant association between visual cross-modal neuroplasticity and benefit from visual cues has been reported in deaf adults (Stropahl et al., 2015; Stropahl and Debener, 2017), our results do not show this same tendency in mild-moderate hearing loss. Based on this finding, it possible that visual cross-modal recruitment of auditory cortex may be related to auditory deprivation itself, rather than enhanced auditory-visual integration, at least in the early stages of hearing loss. This finding is consistent with Stropahl and Debener (2017), where visual (lip-reading) benefit for auditory-visual speech perception was not correlated with strength (amplitude) of visual evoked potential responses to facial stimuli in adults with mild-moderate sensorineural hearing loss.

**FIGURE 7:**
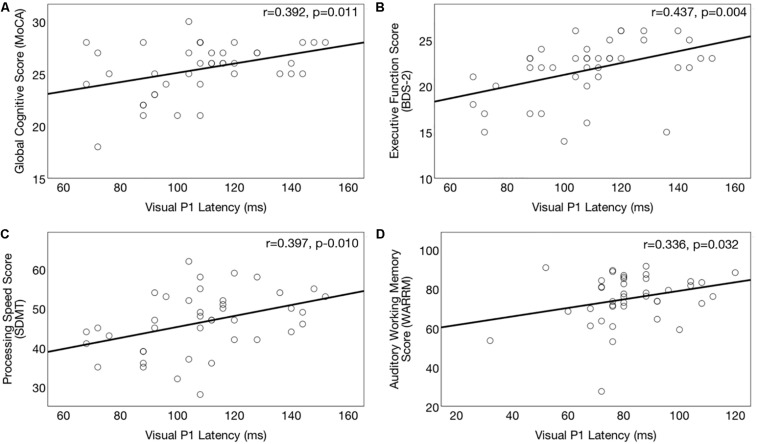
Association between cortical visual evoked potential latencies and cognitive function in the hearing loss group. Significant correlations (*p* ≤ 0.017) between right temporal P1 CVEP peak latency (in milliseconds) and cognitive performance across the domains of global cognitive function on the (Montreal Cognitive Assessment) **(A)**, executive function (Behavioral Dyscontrol Scale) **(B)**, processing speed score (Symbol Digits Modalities Test) **(C)**, and auditory working memory (Word Auditory Recognition and Recall Measure) **(D)**. Higher scores on the cognitive measures indicate better cognitive performance. Earlier CVEP latencies, considered an index of visual cross-modal re-organization, are associated with poorer cognitive functioning.

Correlations between right temporal P1 CVEP latency and performance on the global cognitive function (MoCA), executive function (BDS-2), processing speed (SDMT) and auditory working memory (WARRM) tasks are shown in Figures 7A–D, respectively. Earlier P1 CVEP latency, considered an index of cross-modal re-organization, was associated with poorer global cognitive function (*r* = 0.391, *p* = 0.011) (Figure 7A), executive function (*r* = 0.391, *p* = 0.010) (Figure 7B), processing speed (*r* = 0.397, *p* = 0.010) (Figure 7C), and auditory working memory (*r* = 0.379, *p* = 0.015) (Figure 7D). There was no association between P1 CVEP latency and performance on the visual working memory (RST). The P1 CVEP component is heavily modulated by attention (Hackley et al., 1990; Luck et al., 1990; Gazzaley et al., 2008; Zanto et al., 2010). Thus, it is possible that the correlation between these variables may reflect alterations in top-down modulation of attention. If auditory deprivation induces compensatory changes in visual attention, this may reduce available cortical resources available for other downstream cognitive tasks (Broadbent, 1954; Norman and Bobrow, 1975; Lavie and Tsal, 1994; Lavie, 1995; Rees et al., 1997; Lavie and de Fockert, 2003, 2005; Lavie et al., 2004). While not directly addressed in this study, the unexpected activation of frontal and pre-frontal cortex to visual motion stimuli in the untreated ARHL group (Figure 2) may similarly reflect a shift in attentional and/or cognitive resources for cortical sensory processing in mild-moderate ARHL.

### Effects of Hearing Aid Use on Cortical Visual Evoked Potential Latencies and Amplitudes

**FIGURE 8:**
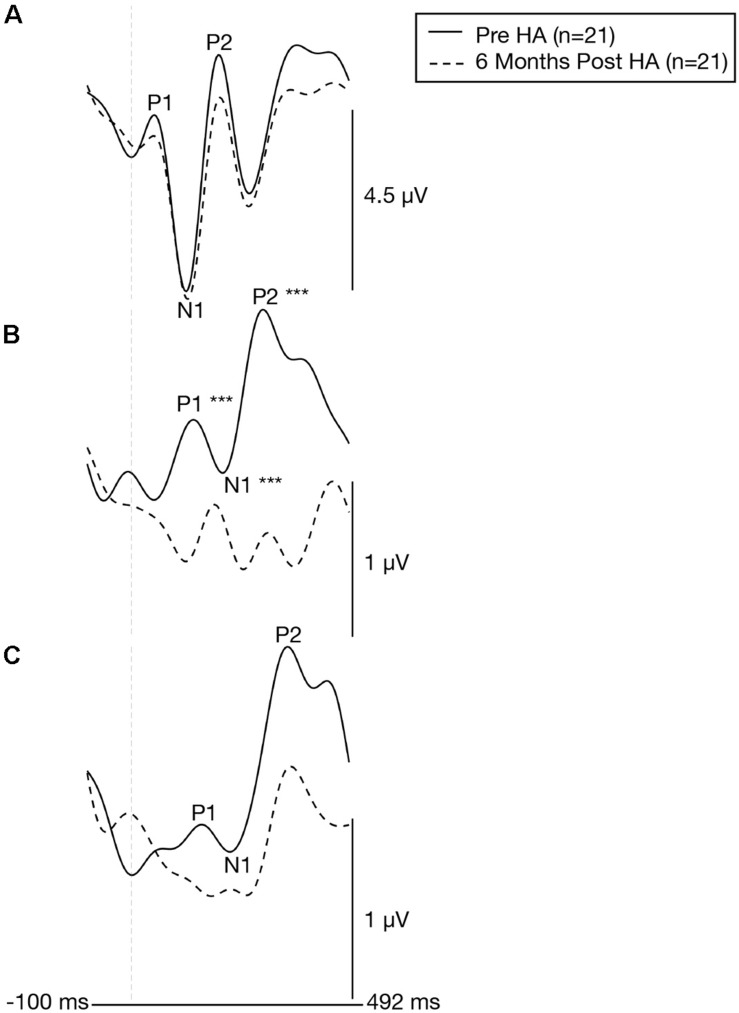
Cortical visual evoked potentials across the occipital, right temporal, and left temporal regions of interest before and 6 months after intervention with hearing aids. Grand-averaged CVEP waveforms for a group of adults with early-stage, age-related hearing loss (*n* = 21) are depicted for the occipital **(A)**, right temporal **(B)**, and left temporal **(C)** regions of interest before (solid black line) and 6 months after hearing aid use (dashed black line). Time (milliseconds) is displayed on the horizontal axis and amplitude (μV) is displayed on the vertical axis. Asterisks indicate level of significance (***p ≤ 0.001). The hearing loss group exhibits a significant late ward shift in CVEP P1, N1, and P2 latencies over the right temporal following treatment with hearing aids.

Plots of the grand average CVEP waveforms for the ARHL group (*n* = 21) at baseline and at 6 months post-treatment follow-up are depicted for the occipital, right temporal, and left temporal ROIs in Figure 8. Table 2 lists the cortical regions of activity for each component in the CVEP response in order of highest to lowest likelihood of activation for the ARHL group pre-treatment and post-treatment. Paired samples *t*-tests indicated no significant treatment effect hearing aid use on P1, N1, or P2 peak latencies or amplitudes over the occipital or left temporal ROIs (*p* > 0.05). However, significant pre-post treatment differences in CVEP P1, N1, and P2 latencies were observed in the right temporal ROI. Specifically, the ARHL group exhibited a significant late-ward shift in post-treatment P1 [*t*(20) = 4.148, *p* < 0.001], N1 [*t*(20) = 5.193, *p* < 0.001], and P2 [*t*(20) = 4.300, *p* < 0.001] CVEP peak latencies with moderate to high effect sizes (Cohen’s *d*-values) [P1: *d* = 0.78, N1: *d* = 1.21, P2: *d* = 0.82]. While average post-treatment amplitudes appear visually reduced, this difference was not statistically significant P1 [*t*(20) = −0.784, *p* = 0.442], N1 [*t*(20) = −0.476, *p* = 0.639], P2 [*t*(20) = −0.460, *p* = 0.650]. To our knowledge, no prior studies have evaluated clinical treatment with hearing aids on visual cross-modal plasticity in ARHL. *Post hoc* group comparisons between the NH group evaluated at baseline (*n* = 13) and the 6 months post-treatment outcomes in the ARHL group (n = 21) indicate no statistical difference in P1 [*t*(32) = 1.339, *p* = 0.190], N1 [*t*(32) = 1.010, *p* = 0.320], or P2 [*t*(32) = 0.814, *p* = 0.422] CVEP latencies over the right temporal ROI, suggesting that restored audibility from hearing aid use may promote more typical cortical visual processing patterns.

**TABLE 2:** Cortical visual evoked potential latencies over a right temporal region of interest in adults with age-related hearing loss before and after 6 months of hearing aid use.

**Component**	**Average latency (ms)**		**Standard deviation**		**95% Confidence interval**		**Statistic *t*(39), (*p*-value)**	**Effect size (Cohen’s *d*)**
	**Pre-HA**	**Post-HA**	**Pre-HA**	**Post-HA**	**Pre-HA**	**Post-HA**		
P1	101	118	19.4	23.78	92–110	108–129	4.15 (<0.001)	0.78
N1	133	166	26.9	27.75	121–145	154–179	5.19 (<0.001)	1.21
P2	196	231	41.18	44.55	178–218	212–252	4.30 (<0.001)	0.82

**Average peak latencies, standard deviations, 95% confidence intervals, statistical significance values, and effect size are provided for the age-related hearing loss group pre-treatment and 6 months post-treatment (n = 21). A significant late-ward shift in P1, N1, and P2 latencies are observed with hearing aid use.**

### Effects of Hearing Aid Use on Cortical Visual Evoked Potential Current Density Source Reconstruction Patterns

**FIGURE 9:**
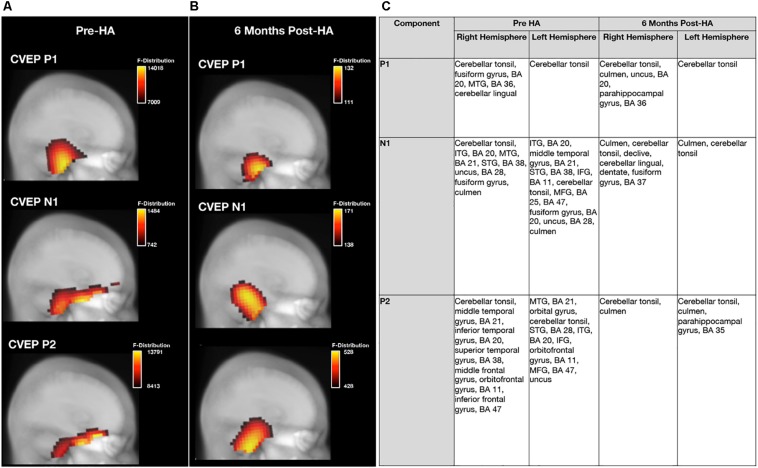
Effects of treatment with hearing aids on cortical source activation patterns elicited by visual motion stimuli in age-related hearing loss. **(A)** Depicts current density source reconstructions (CDR) for the P1, N1, and P2 cortical visual evoked potential (CVEP) components for the group of adults with untreated age-related hearing loss assessed at baseline prior to hearing aid fitting (pre-HA) (*n* = 28) and **(B)** depicts average CDRs for the P1, N1, and P2 CVEP components in a sub-group of these adults (*n* = 21) assessed post-treatment after 6 months of hearing aid use (post-HA). 3D current density source reconstructions obtained via standardized low-resolution brain electromagnetic tomography (sLORETA) are projected on a 2D depth-buffered structural magnetic resonance image (Maximum Intensity Projection), providing visualization of the voxels with the highest likelihood of activation. The color scale to the right of each figure indicates the statistical likelihood of activation (F-statistic), from lowest (red) to highest (yellow) probable current density. **(C)** Describes regions of cortical source activity for each CVEP component, including Brodmann areas (BA), for the age-related hearing loss group at baseline and 6-month follow-up by rank in order highest to lowest likelihood of activation (F-statistic).

Pre-treatment and 6 months post-treatment CVEP CDRs for the ARHL group are displayed in Figure 9. Please note that since stability of cortical sources localization (and SNR) increases with larger subject numbers, all ARHL subjects who were assessed at baseline (*n* = 28) were compared to the group of ARHL adults who met minimum hearing aid usage criterion 6 months post-treatment with hearing aids (*n* = 21). While the ARHL exhibited occipital, temporal (e.g., superior, middle, and inferior temporal gyrus), and frontal and pre-frontal cortical activity [e.g., orbitofrontal gyrus, Brodmann area (BA) 11] for the higher-order N1 and P2 CVEP components pre-treatment, there was a post-treatment reduction in auditory cortex recruitment for these components post-treatment, suggestive of a reversal in visual cross-modal re-organization by vision. In addition, post-treatment results indicate a reduction in frontal and pre-frontal cortex activation compared to baseline. Post-treatment CDR results in the hearing loss group are comparable those results observed in the NH group at baseline evaluation (Figure 3A).

### Effects of Hearing Aid Use on Speech Perception in Noise

**FIGURE 10:**
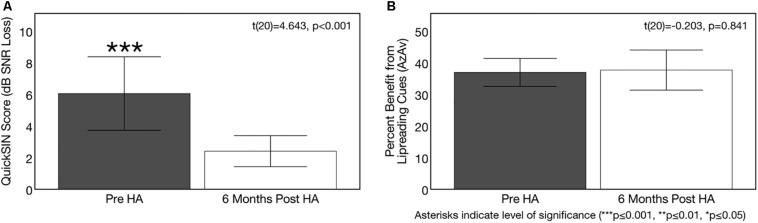
Effects of treatment with hearing aids on auditory speech perception in noise. Average scores on speech perception measures in the age-related hearing loss group pre-treatment (gray) and 6 months post-treatment with hearing aids (Post-HA) (white). Black bars indicate 95% confidence intervals for each group. Asterisks indicate level of significance (***p ≤ 0.001, **p ≤ 0.01, *p ≤ 0.05). **(A)** Depicts average binaural auditory speech perception in noise scores (QuickSIN^TM^) before and after hearing aid treatment. Scores are plotted in terms of the dB signal-to-noise ratio loss (dB SNR), representing the dB SNR required for the participant to score 50% of words in a sentence correct relative to normal hearing listeners. A higher score indicates poorer auditory speech perception in noise performance. The dotted line indicates the cutoff score (3 dB SNR) for normal function in background noise. The hearing loss group exhibited significant improvements in auditory speech perception in noise after treatment with hearing aids. **(B)** Shows average benefit from visual (lip-reading) cues on a binaural auditory-visual speech perception in noise test (AzAv). Scores indicate the percent difference score in an auditory-only condition relative to an auditory-visual condition, where a higher score indicates greater benefit from the addition of visual (lip-reading) cues. No significant difference in lip-reading benefit was observed between pre-post intervention test sessions in the hearing loss group.

Figure 10A depicts auditory speech perception in noise scores and corresponding 95% confidence intervals in the ARHL group pre-treatment and post-treatment with hearing aids. A significant pre-post treatment improvement in QuickSIN^TM^ score was observed [*t*(20) = 4.643, *p* < 0.001]. While ARHL adults exhibited a mild auditory deficit (3–7 dB SNR) in background noise without hearing aids (average score = 6.05 dB SNR, *SD* = 5.11), treatment with hearing aids over yielded a 3.6 dB SNR improvement in performance (average score = 2.40 dB SNR, *SD* = 2.15), with performance comparable to NH adults (0–3 dB SNR).

Figure 10B depicts sentence-level visual (lip-reading) benefit for auditory-visual speech perception in noise (AzAv) at baseline and 6 months follow-up in the ARHL group, as well as corresponding 95% confidence intervals. The ARHL group performed derived similar benefit from visual cues on the AzAv test pre- and post-treatment with hearing aids [*t*(20) = −0.203, *p* = 0.841]. Average benefit from visual cues pre-treatment at the baseline evaluation (acutely aided condition) was 36.93% (*SD* = 9.69) and average benefit from visual cues at 6 months post-treatment follow-up (aided condition) was 37.66% (*SD* = 13.92). This finding suggests that hearing aid use does not modify auditory-visual integration in mild-moderate ARHL. However, given that adults with hearing loss did not have an advantage in lip-reading at the pre-treatment baseline compared to NH adults, it was not entirely unexpected that there would be no change in their results after treatment. No correlation was observed between average daily hearing aid use and change in auditory speech perception in noise performance (QuickSIN^TM^) [*t*(20) = −0.148, *p* = 0.523] or change in dependence on visual cues for auditory-visual speech perception in noise AzAv test: [*t*(20) = −0.210, *p* = 0.362]. Given high homogeneity of average daily hearing aid use among ARHL participants (average = 9.84 h/day, *SD* = 2.96, range = 5.10–14.02 h/day), this is not an unexpected finding.

### Effects of Hearing Aid Use on Cognitive Function

**FIGURE 11:**
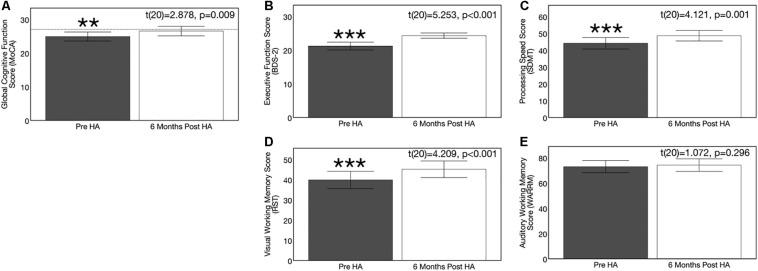
Effects of treatment with hearing aids on cognitive function in age-related hearing loss across 6 months of hearing aid use. Average scores on cognitive measures in the age-related hearing loss group (*n* = 21) are depicted pre-treatment (gray) and 6 months post-treatment with hearing aids (white). Black bars indicate 95% confidence intervals for each group. Asterisks indicate level of significance (***p ≤ 0.001, **p ≤ 0.01, *p ≤ 0.01). **(A)** Shows average global cognitive function on a global cognitive screening measure (MoCA) pre- and post-treatment. Higher scores in indicate higher global cognitive function, out of a total score of 30 points. The dotted reference line on the y-axis indicates the cutoff score (≤ 27) indicating risk for mild cognitive impairment. A significant improvement in MoCA score was observed post-treatment in the hearing loss group. **(B)** Depicts average executive function score (BDS-2) pre- and post-treatment. Higher scores in indicate better executive functioning, out of a total score of 27 points. A significant improvement in executive function score was observed post-treatment in the hearing loss group. **(C)** Depicts average processing speed score (SDMT) pre- and post-treatment. Higher scores in indicate faster processing speeds in a timed, 90 s digit-symbol matching task A significant improvement in processing speed was observed post-treatment in the hearing loss group. **(D)** Shows average visual working memory score (RST) in percent words correctly recalled pre- and post-treatment. Higher scores indicate higher visual working memory performance in a dual-task paradigm. A significant improvement in visual working memory recall was observed post-treatment in the hearing loss group. **(E)** Depicts average auditory working memory score (WARRM) in percent words correctly recalled pre- and post-treatment. Higher scores indicate higher auditory working memory performance in a dual-task paradigm. No significant improvement in auditory working memory recall was observed post-treatment in the hearing loss group. Note: Cognitive testing in the hearing loss group was administered in the same condition across pre-treatment (acutely aided) and post-treatment (aided) test sessions to ensure similar pre-posttest conditions and to reducing potential confounding effects of audibility on cognitive performance at the pre-treatment visit.

Hearing aid use over the course of 6 months resulted in significant improvements in the domains of global cognitive function, executive function, processing speed, and visual working memory (but not auditory working memory). On the global cognitive function measure (MoCA), 71% (*n* = 15) of ARHL adults showed improved performance after 6 months of hearing aid use, 10% (*n* = 2) showed no change in performance, and 19% (*n* = 4) showed decreased performance. On the executive function measure (BDS-2), 90% of subjects (*n* = 19) showed improvement performance, 10% showed no change in performance (*n* = 2), and 0% (*n* = 0) showed decreased performance. On the processing speed measure (SDMT), 81% of subjects (*n* = 17) showed improved performance, 5% showed no change in performance (*n* = 1), and 14% (*n* = 3) showed decreased performance. On the visual working memory test (RST), 71% showed improved performance (*n* = 16), 0% showed no change in performance, and 24% showed decreased performance (*n* = 5). On the auditory working memory test (WARRM), 67% showed improved performance (*n* = 14), 0% (*n* = 0) showed no change in performance, and 33% showed decreased performance (*n* = 7). Pre-post cognitive test results on these cognitive assessments are depicted in Figures 11A–E. A 1.62 point improvement in global cognitive function (MoCA) score was observed after 6 months of hearing aid use compared to pre-treatment, which was statistically significant [*t*(20) = 2.878, *p* = 0.009]. Average improvement in executive function (BDS-2) after 6 months of hearing aid use was 3.09 points higher than pre-treatment scores, and this improvement was also significant [*t*(20) = 5.253, *p* < 0.001]. Significant improvements in processing speed (SDMT) by 4.52 points [*t*(20) = 4.209, *p* < 0.001] and visual working memory (RST) by 5.30 percentage points [*t*(20) = 4.121, *p* = 0.001] were also observed after 6 months of hearing aid use. We observed no significant improvement on the auditory working memory (WARRM) measure following treatment with hearing aids [*t*(20) = 1.072, *p* = 0.296].

Further, *post hoc* correlational analyses indicate that reliance on cognitive function is greater in situations where the acoustic speech signal is unfavorable (e.g., unaided) compared to situations where the acoustic speech signal is more optimal (e.g., appropriately aided). For example, the correlations between unaided auditory speech perception in noise (QuickSIN^TM^ score) and performance on the global cognitive function task (MoCA) (*r* = −0.37, *p* = 0.018) and processing speed task (SDMT) (*r* = −0.427, *p* = 0.005) measured at baseline were stronger than the correlations between aided auditory speech perception in noise (QuickSIN^TM^ score) and performance on the global cognitive function task (MoCA) (*r* = −0.446, *p* = 0.043) and processing speed task (SDMT) (*r* = −0.292, *p* = 0.199) measured 6 months after hearing aid use. This finding is consistent with previous studies which show that acoustically degraded speech requires greater cognitive compensation (Rönnberg et al., 2013, 2008; Wingfield et al., 2015). No correlation was observed between average daily hearing aid use and change performance on any of the cognitive tasks (MoCA: [*t*(20) = 0.046, *p* = 0.843]; BDS-2: [*t*(20) = −0.11, *p* = 0.618]; SDMT: [*t*(20) = 0.260, *p* = 0.254]; RST: [*t*(20) = 0.143, *p* = 0.535]; WARRM: [*t*(20) = 0.355, *p* = 0.114]). Given high homogeneity of average daily hearing aid use among ARHL participants (average = 9.84 h/day, *SD* = 2.96, range = 5.10–14.02 h/day), this is not an unexpected finding.

### Pre-treatment Cross-Modal Re-organization Predicts 6 Months Post-treatment Auditory Speech Perception Outcomes

**FIGURE 12:**
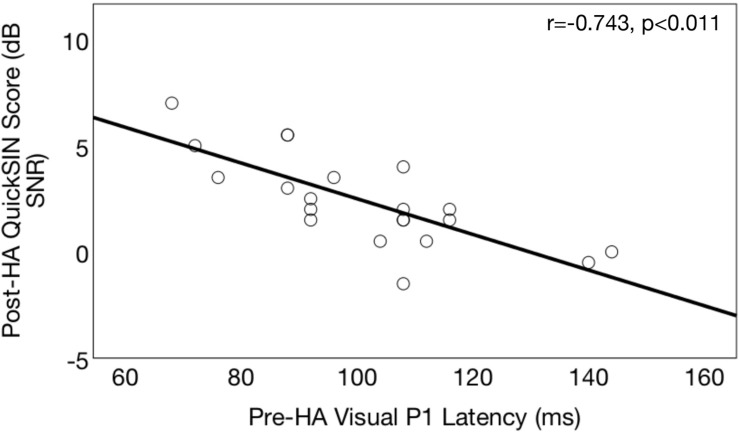
Association between pre-treatment cortical visual evoked potential latencies and post-treatment auditory speech perception in noise outcomes in age-related hearing loss. Correlations between pre-treatment neurophysiological outcomes and post-treatment speech perception outcomes are shown for a group of adults with hearing loss (*n* = 21) who received treatment with hearing aids. Pre-treatment right temporal P1 CVEP peak latency (in milliseconds) is displayed on the horizontal axis and performance 6 months post-treatment binaural aided auditory speech perception in noise performance (QuickSIN^TM^ score) is depicted on the vertical axis. QuickSIN^TM^ scores are plotted in terms of the dB signal-to-noise ratio loss (dB SNR), representing the dB SNR required for the participant to score 50% of words in a sentence correct relative to normal hearing listeners. A higher score indicates poorer auditory speech perception in noise performance. Earlier pre-treatment P1 CVEP latencies (an index of visual cross-modal re-organization) are predictive of poorer post-treatment auditory outcomes.

Figure 12 shows the correlation between pre-treatment CVEP latencies and post-treatment QuickSIN^TM^ scores in the ARHL group. As shown, there was a significant negative correlation was observed between pre-treatment CVEP latencies in the right temporal ROI and auditory speech perception in noise outcomes for the P1 (*r* = −0.743, *p* < 0.001), N1 (*r* = −0.643, *p* < 0.001), and P2 (*r* = −0.532, *p* < 0.001) components, suggesting that the cross-modal state of the auditory cortex pre-treatment is predicted of 6 months post-treatment auditory speech perception outcomes. No such association was observed between pre-treatment right temporal CVEP latencies and post-treatment dependence on visual (lip-reading cues) or post-treatment cognitive outcomes across the domains of global cognitive function, executive function, processing speed, auditory working memory, or visual working memory.

Tinnitus status in the ARHL group had no effect on performance outcomes on the QuickSIN^TM^ [*t*(20) = 1.027, *p* = 0.318], AzAv [*t*(20) = 0.583, *p* = 0.567] or on majority of the cognitive tests (MoCA, BDS-2, RST, WARRM) [*t*(20) < 1.659, *p* > 0.114]. Gender had no effect on performance outcomes on the QuickSIN^TM^ [*t*(20) = 0.814, *p* = 0.426], AzAv [*t*(20) = 0.175, *p* = 0.062], or majority of the cognitive tests (BDS-2, SDMT, WARRM) [*t*(20) < 0.175, *p* > 0.863], though females performed slightly better than males on the global cognitive function test (MoCA) [*t*(20) = 2.104, *p* = 0.049] and visual working memory test (RST) [*t*(20) = 2.432, *p* = 0.030] post-treatment. Age was not correlated with performance outcomes on the QuickSIN^TM^ (*r* = −0.007, *p* = 976), AzAv (*r* = −0.012, *p* = 0.959), or majority of the cognitive tests (MoCA, SDMT, RST, WARRM) (*r* < 0.358, *p* > 0.111) in the ARHL group, though older age was correlated with poorer executive function (BDS-2) (*r* = −0.645, *p* = 0.002).

### Subjective Self-Report of Hearing Aid Benefit and Satisfaction

**FIGURE 13:**
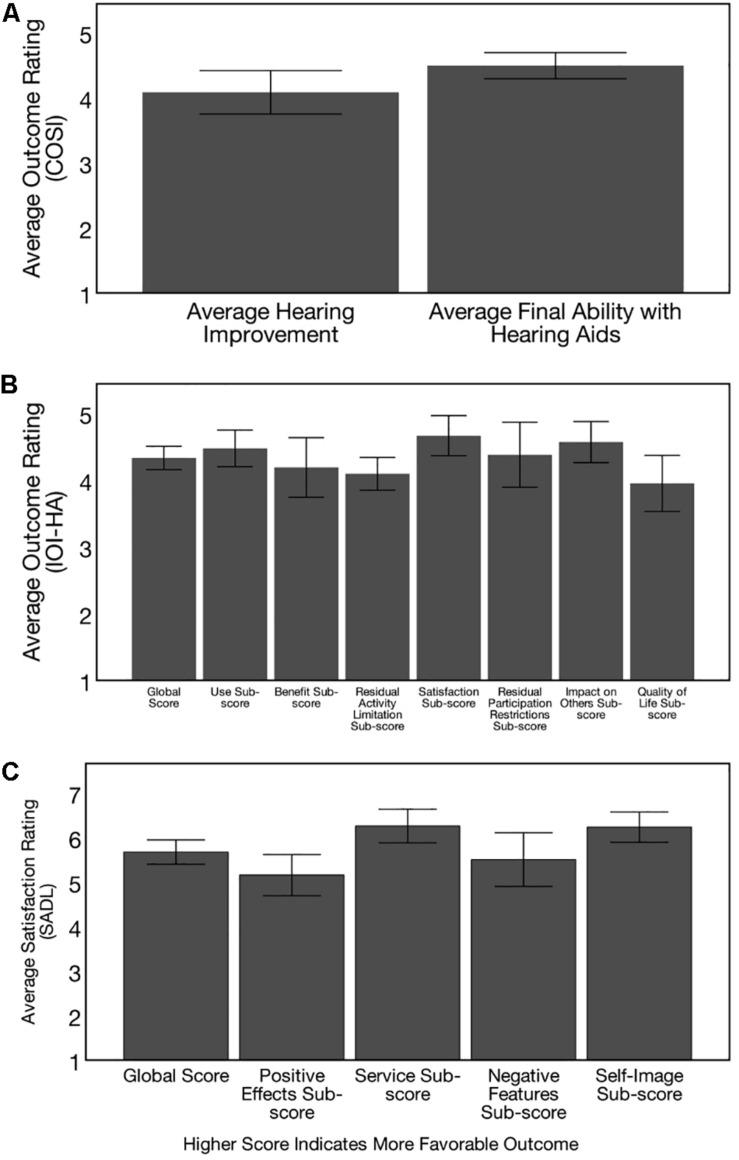
Post-treatment hearing aid outcomes. Outcomes on 3 self-report questionnaires are depicted, with higher scores indicating more favorable outcomes for the adults with hearing loss (*n* = 21) who received treatment with hearing aids. **(A)** Depicts outcomes on the Client Oriented Scale of Improvement (COSI), where subjects were asked to identify 5 specific listening situations where they wanted to see improvement with hearing aids. Six months post-treatment, participants were asked to rate improvement with hearing aids in each of these situations on a 5-point scale (1 = worse, 2 = no difference, 3 = slightly better, 4 = better, and 5 = much better) and their final hearing ability to hear with hearing aids in each of these situations on a 5-point scale (1 = hardly ever, 2 = occasionally, 3 = half the time, 4 = most of the time, and 5 = almost always). Hearing loss participants exhibit improvements with hearing aid use in the listening situations identified as most important to them and can hear “most of the time” (> 75%) in the listening situations identified as most important to them. These results provide subjective data of benefit from clinical treatment of mild-moderate, age-related hearing loss. **(B)** Shows hearing aid Outcomes on the International Inventory of Hearing Aids (IOI-HA), where subjects provided a rating for 7-items assessing daily use of hearing aids, benefit, residual activity limitations, satisfaction, residual participation restrictions, impact on others, and quality of life at the 6 months post-treatment visit. Average global improvement rating was 4.33 (*SD* = 0.38) and sub-scores were also high, providing subjective evidence of benefit from hearing aid treatment. **(C)** Shows hearing aid outcomes on the Satisfaction with Amplification in Daily Life Questionnaire (SADL), where subjects were asked to provide a rating for 15 items assessing positive effects of hearing aid use, service, negative features, and personal image on a 7-point scale at the 6 months post-treatment follow-up. Average global score was 5.68 (*SD* = 0.60) and sub-scores were also high, indicating high levels of self-perceived satisfaction with hearing aids in early-stage, mild-moderate age-related hearing loss.

Self-report of hearing aid benefit and satisfaction on the COSI, IOI-HA, and SADL is depicted in Figures 13A–C, respectively. On the COSI outcome questionnaire (Figure 13A), subjects were asked to identify several listening situations they identified as most important to them at the baseline evaluation, and then were asked to rate their improvement with hearing aids and their final ability with hearing aids in these specific situations on a 5-point scale, with a higher score indicating greater levels of improvement. Average improvement rating with hearing aids was 4.09 (out of 5) (*SD* = 0.60) and average final ability with hearing aids 4.49 (*SD* = 0.44) (out of 5) on the COSI outcome questionnaire, indicating the ARHL group felt they were able to hear most of the time (>75%) with their hearing aids in the specific listening situations they identified as most important to them.

On the IOI-HA outcome questionnaire, ARHL subjects were asked to provide a rating for 7-items assessing daily use of hearing aids, benefit, residual activity limitations, satisfaction, residual participation restrictions, impact on others, and quality of life at the 6 months follow-up visit. Ratings were provided on a 5-point scale, where a lower score indicates poorer outcome and a higher score indicates higher outcome for each item. Average global improvement rating was 4.33 (*SD* = 0.38), indicating significant benefit from hearing aid use (Figure 13B). An additional 8th item on the IOI-HA test probed subject’s self-reported hearing difficulty on a 5-point scale (1 = severe, 2 = moderately-severe, 3-moderate, 4 = mild, 5 = none), where a higher score indicates less self-perceived difficulty. Based on the results, the average self-reported difficulty on item 8 of the IOI-HA was 3.19 (*SD* = 0.75), indicative of moderate self-reported hearing difficulty in background noise. Average global score across items 1–7 on the IOI-HA was 4.33 (*SD* = 0.38). Average scores across items 1–7 were 4.47 (= 0.60) for the use sub-score, 4.19 (*SD* = 0.99) for the benefit sub-score, 4.10 (*SD* = 0.54) for the residual activity limitation sub-score, 4.67 (*SD* = 0.67) for the satisfaction sub-score, 4.38 (*SD* = 1.07) for the participation sub-score, 4.57 (*SD* = 0.68) for the impact on others sub-score, and 3.95 (*SD* = 9.92) for the quality of life sub-score. These results were compared to normative data in adults with moderate-severe sensorineural hearing loss reporting “moderate” hearing problems on item 8 reported in Cox et al. (2003). Results from our study subjects are comparable these norms across all 7 sub-scores, even though our study subjects had slightly less severe hearing loss. Results provide evidence of real-world effectiveness of hearing aid intervention in the mild-moderate stages of ARHL.

On the SADL outcome measure, the ARHL group was asked to indicate their relative satisfaction with hearing aids across the areas of positive effect, service, negative features, and personal image (Figure 13C). Higher scores indicate greater self-perceived satisfaction. Average global score on the SADL was 5.68 (*SD* = 0.60). Average positive effect sub score was 5.15 (*SD* = 1.02), average service sub-score was 6.26 (*SD* = 0.83), average negative feature sub-score was 5.51 (*SD* = 1.32), and average self-image sub-score was 6.24 (*SD* = 0.75). Comparison of our results against normative data reported in Cox and Alexander (2001) indicate global scores and sub-scores falling above the 50th percentile, and service sub-score and negative features sub-scores falling above the 80th percentile for adults with similar degree of hearing loss. Together, results from these hearing aid outcome measures provide evidence of high levels self-perceived benefit and satisfaction from hearing aids in our study sample.

## Discussion

Overall, the goal of this investigation was to explore the relationship between cortical visual cross-modal neuroplasticity and speech perception and cognitive outcomes in early-stage ARHL, and to assess treatment effects with well-fit hearing aids on these outcomes. Visual cross-modal re-organization was observed in the untreated ARHL group, as evidenced by earlier CVEP latencies over right auditory cortex and cortical source localization patterns indicating greater probable current densities in auditory cortex to visual motion stimuli. Visual cross-modal re-organization in the ARHL group was associated degree of hearing loss and poorer auditory speech perception outcomes, but not visual (lip-reading) benefit. More extensive cross-modal re-organization in the ARHL group at baseline was also associated with poorer cognitive performance in the domains of global cognitive function, executive function, processing speed, and auditory and visual working memory. As a group, clinical treatment with well-fit amplification reversed cross-modal recruitment of auditory cortex for visual processing in the ARHL group following 6 months of hearing aid use, coinciding with gains in auditory speech perception abilities and improvements in global cognitive function, executive function, processing speed, and visual working memory performance. Further, the cross-modal status of the right auditory cortex at baseline before hearing aid fitting was predictive of 6 months post-treatment auditory speech perception outcomes. To our knowledge, this is the first study to document reversal in visual cross-modal re-organization following clinical intervention with hearing aids, though reversal in cross-modal re-organization has been previously reported in an individual pediatric case of single-sided deafness following clinical intervention with a cochlear implant (Sharma et al., 2016).

### Cortical Visual Cross-Modal Neuroplasticity in Mild-Moderate Age-Related Hearing Loss

A main finding from this study was more extensive cross-modal recruitment of auditory cortex by vision in the ARHL group prior to hearing aid fitting. We observed earlier P1, N1, and P2 latencies in the ARHL group relative to NH controls over the right temporal cortex at baseline, and CVEP current density source reconstruction patterns indicating greater cross-modal activity over the auditory cortex. This finding replicates results from a previous high-density EEG study in a smaller group of adults with mild-moderate ARHL using the same stimulus (Campbell and Sharma, 2014) as well as other EEG studies using different visual stimuli (Stropahl and Debener, 2017). Earlier CVEP latencies have been reported in deaf adults and adults with ARHL in previous EEG studies, and are considered an index of visual cross-modal re-organization (Neville and Lawson, 1987; Finney et al., 2003; Fine et al., 2005; Doucet et al., 2006; Buckley and Tobey, 2011; Sandmann et al., 2012; Hauthal et al., 2013; Campbell and Sharma, 2014), where earlier latencies reflect increased synaptic strength and connectivity (Driver and Spence, 2004). Our observations of more extensive visual cross-modal re-organization in the right temporal ROI is also similar to findings from Cardon and Sharma (2018), where there was more extensive cross-modal recruitment of right auditory cortex by the somatosensory modality in adults with ARHL compared to NH controls. Because right auditory cortex has been shown to be more susceptible to atrophy in ARHL (Lin et al., 2014), the deprived auditory cortex may be recruited or ‘re-purposed’ for visual or somatosensory processing. While this phenomenon was once believed to restricted to severe-profound hearing loss (e.g., deafness), our results support a growing body of evidence that even mild auditory deprivation may induce compensatory changes in cortical neuroplasticity (Campbell and Sharma, 2014; Stropahl and Debener, 2017).

### Mechanisms of Cortical Visual Cross-Modal Neuroplasticity in Adult-Onset Hearing Loss

Second, results from this study indicate that untreated mild-moderate ARHL is associated with deficits in auditory speech perception in noise and cognitive functioning. The untreated ARHL group exhibited significantly poorer cognitive performance across the domains of global cognitive function, executive function, processing speed, visual working memory, and auditory working memory compared to the NH group at baseline. Poorer global cognitive outcomes in ARHL have been reported in previous cross-sectional studies (Lindenberger and Baltes, 1994; Baltes and Lindenberger, 1997; Gussekloo et al., 2005; Lin et al., 2011, 2013; Deal et al., 2015; Dupuis et al., 2015; Harrison Bush et al., 2015) and cohort studies (Gallacher et al., 2012; Lin et al., 2013; Deal et al., 2015; Hong et al., 2016). Impairments on measures of executive functioning have been previously reported in ARHL cohorts (Gates et al., 1996, 2010; Lin et al., 2013). ARHL is also associated with slower processing speeds (Clark, 1960; Anstey et al., 2001; Valentijn et al., 2005; Lindenberger and Ghisletta, 2009; Lin, 2011; Lin et al., 2011, 2013; Gallacher et al., 2012; Deal et al., 2015, 2017; Bucks et al., 2016) and deficits in working memory (Anstey and Smith, 1999; Hofer et al., 2003; MacDonald et al., 2004; Harrison Bush et al., 2015; Bucks et al., 2016) relative to NH adults. In our study auditory speech perception and cognitive performance was significantly associated with visual cross-modal re-organization, such that earlier latencies (considered a marker of visual cross-modal recruitment of auditory cortex for visual processing) was associated with poorer auditory speech perception and cognitive performance. This finding is consistent with the cognitive load theory (Pichora-Fuller et al., 2016), whereby decreased audibility and/or degraded auditory input in hearing loss taxes the brain, resulting in increased cognitive load, depleting spare capacity for other tasks such as memory.

The pre-frontal and frontal cortex recruitment for visual processing that was observed in the ARHL group at baseline – which was absent in the NH group – is a new and unexpected finding. Previous studies have found that hearing impaired listeners may exhibit greater frontal cortex activity when processing incongruent audio-visual, auditory, and visual speech stimuli (McGurk Effect), which is presumed to reflect an increase in cognitive effort during auditory-visual integration tasks (Rosemann and Thiel, 2018). Recruitment of frontal and pre-frontal cortex has also been reported in hearing impaired listeners under difficult auditory speech perception tasks such as in background noise (Obleser et al., 2007; Wong et al., 2009). It is also possible that the pre-frontal and frontal cortex activity observed in our ARHL group at baseline may reflect changes in top-down modulatory control. For example, functional interactions between the pre-frontal and visual cortex have been shown to contribute enhance visual processing, and it is presumed that this modulation by prefrontal cortex may enhance visual attention (Gazzaley et al., 2007). Similarly, pre-frontal cortex appears to modulate auditory cortex during speech processing tasks, with more pronounced effects in left compared to right auditory cortex (Park et al., 2015). Thus, hearing loss may alter normal top-down modulatory control by pre-frontal and frontal cortex of sensory cortices. The reduction in frontal and pre-frontal cortex activity in the ARHL post-treatment suggests that hearing aid use may reduce cognitive effort and/or alter top-down modulation of auditory or visual cortex for visual processing.

### Potential for Hearing Aid Use to Reverse Visual Cortical Cross-Modal Re-organization and Provide Cognitive Benefit

Notably, as a group our ARHL subjects showed a reversal in visual cross-modal recruitment of auditory cortex within 6 months of hearing aid use. Moreover, this reversal in cross-modal re-organization coincided with recovery in auditory speech perception in noise performance. Performance on cognitive measures in the ARHL group also improved 6 months post-treatment in almost all cognitive sub-domains (global cognitive function, executive function, processing speed, and visual working memory) except for auditory working memory, where test performance approximated performance of the NH control group at baseline. Thus, beyond the known benefits hearing aid use in improving speech perception and communication, our results provide preliminary evidence that hearing aid use may enhance cognitive function.

It is important to emphasize that cognitive assessment at baseline and 6 months follow-up was performed in an aided condition for the ARHL group, reducing the potential confound of audibility on pre-post treatment differences. This study lacked a control group at 6 months follow-up, in order to mitigate this to some extent, we sought to use best clinical practices by choosing cognitive measures with good test-retest reliability over short test intervals. The global cognitive function measure (MoCA) show high test-retest reliability (*r* = 0.96) with re-test occurring 2 weeks apart (Wong et al., 2009), with slightly lower test-retest reliability (*r* = 0.75–0.92) over a range of 4-8 weeks or longer (Lee et al., 2008). Test-retest reliability for the executive function measure (BDS-2) is high (*r* = 0.8) at 8 week and 6 months follow-up intervals (Grigsby et al., 1992, 2002a,b). Test-retest reliability of the SDMT is high (*r* = 0.7–0.9) when administered over the course of 2 weeks, 1 month, or 2 years intervals (Benedict et al., 2017). High test-retest reliability over minutes (Turley-Ames and Whitfield, 2003), weeks (Klein and Fiss, 1999; Friedman and Miyake, 2004), and months (Klein and Fiss, 1999) is reported for the visual working memory test (RST). High intra-session and inter-session and test-retest reliability (*r* > 0.8) has also been reported for the auditory working memory measure (WARRM) (Smith et al., 2016). However, we cannot rule out potential practice effects since we were unable to test the NH subjects at 6 months.

It is possible that reversal in visual cross-modal neuroplasticity in ARHL may provide an objective marker of treatment benefit. For example, less diffuse cross-modal re-organization has also been reported in deaf adults with good auditory speech perception outcomes following cochlear implantation, while deaf adults with poor auditory outcomes exhibit persistent cross-modal re-organization that persists even years after cochlear implantation (Doucet et al., 2006).

To our knowledge, this is the first study to provide evidence that restored audibility with hearing aids may reverse compensatory changes in cortical resource allocation and promote typical more typical visual sensory processing patterns, coinciding with speech perception benefit and cognitive gains, though other studies indicate neurocognitive benefit from hearing aid treatment in adults with hearing loss. For example, findings from a recent study by Anderson (2019) revealed measurable improvements in working memory after 6 months of hearing aid use in a group of adults with hearing loss relative to NH controls (who showed no change in cognitive performance). Further, cognitive gains in their hearing loss group were associated with P2 cortical auditory evoked potential (CAEP) amplitudes, suggesting that increased auditory input may provide neurocognitive benefit. Our findings are also supported by experimental evidence by Deal et al. (2017) and Karawani et al. (2018a), where hearing aid treatment over longer durations (>6 months) in similar ARHL populations resulted in significant improvements in cognitive function the cognitive domains of global cognitive abilities and processing speed (Deal et al., 2017) as well as improvements in working memory (Karawani et al., 2018b). In addition, long-term neurocognitive benefit has been reported in deaf adult cochlear implant recipients at 6 months and 1 year post post-treatment, where notable gains were observed in the areas of global cognitive function and executive function (Mosnier et al., 2015). These results are in contrast to Nkyekyer et al. (2019), where researchers found no improvement in cognitive function in a group of adults with hearing loss (*n* = 40) fit with hearing aids and examined over the course of 6 months. However, different cognitive sub-domains were assessed in this study (reaction times, immediate and delayed recall, spatial working memory, and contextual memory), study subjects were almost a decade older than the subjects in our study, and there was limited information provided with regards to the quality of the hearing aid fitting or the duration of hearing aid use during the study. The potential for hearing aid use to provide cognitive benefit may depend on a variety of factors (e.g., age, duration of hearing loss, quality of hearing aid fitting, hearing aid use) or take longer in some patients. Future studies should seek to understand this relationship. It is possible that audiological intervention may only provide neurocognitive benefit if treatment is delivered in a timely manner, before extensive functional and structural neural changes take place.

Interestingly, the extent of visual cross-modal re-organization of auditory cortex pre-treatment (as indexed by earlier cortical visual evoked potential latencies) was predictive of auditory speech perception in noise outcomes 6 months post-treatment. This finding suggests that there may be an upper limit to reversing compensatory changes in cortical resource allocation. That is, recovery of auditory speech perception abilities after clinical intervention may be limited by the extent to which auditory cortex has become “re-purposed” by vision. For the small percentage of adults with ARHL who do seek treatment, treatment is typically sought out late, delayed 7–10 years after the hearing loss onset (Davis et al., 2007). Thus, audiological intervention with hearing aids is likely introduced to a central auditory system that has extensively re-organized after long periods of deprivation, potentially limiting treatment effects of hearing aid or cochlear implants. It is possible that these alterations in visual cross-modal re-organization (or other changes in cortical resource allocation) may contribute to the wide variability in outcomes observed in adults with hearing loss who receive treatment. Extensive central “re-wiring” of the auditory pathways could explain low levels of uptake and use with hearing aids.

### Future Directions

This study was not a randomized controlled clinical trial and the NH subjects were not re-tested at 6 months follow-up. At baseline, the untreated ARHL group and NH control group demonstrated clear differences in CVEP latencies, cortical source activation patterns, and cognitive performance. After 6 months of hearing aid use, CVEP latencies significantly increased (right auditory ROI), cortical source activation patterns showed less extensive cross-modal re-organization, and cognitive performance improved in the ARHL group, with measures similar to the NH group at baseline. While preliminary findings from this study supports the idea that early and timely intervention with hearing aids (e.g., in the mild-moderate stages) may provide the best chance of promoting typical cortical sensory functioning and good prognostic cognitive and behavioral outcomes, future randomized controlled trials can provide more robust evidence regarding cortical and cognitive benefit from hearing aid treatment.

Longitudinal follow-up studies are also necessary to understand whether extended hearing aid use (beyond 6 months as reported in our study and previous studies) may modify long-term risk for cognitive decline, including dementia. Currently, there exists no universal screening of hearing loss in the United States and very few adults with clinically significant ARHL use hearing aids or cochlear implants. Research in this area may lead to better tools to diagnose auditory deprivation in its early stages and may also help target optimal timeframes for intervention.

It is important to note that ARHL subjects in our study were fit with well-fit hearing aids (± 5 dB of NAL-NL2 targets measured using probe-microphone measures). ARHL subjects in our study also complied with a high level of daily hearing aid use greater (average = 9.84 h/day, *SD* = 2.96). It is possible that poorly fit devices or low levels of compliance may reduce the efficacy of hearing aids in providing benefit. Future studies should seek to understand how the quality of hearing aid fitting and/or amount of daily hearing aid use may influence auditory, cortical, cognitive outcomes following intervention. Further, while this study focused on group-level differences in baseline auditory, cortical, and cognitive function in NH adults and adults with ARHL, future studies should seek to understand differences in individual characteristics and demographic variables that may affect cortical and cognitive outcomes following intervention for adults with ARHL. Such information may help inform best-practice guidelines and help guide clinical recommendations.

ARHL subjects in our study were fit with hearing aids but received no additional rehabilitation services beyond intervention with hearing aids. Future studies should evaluate whether intervention coupled with additional rehabilitation (e.g., auditory training) may help maximize auditory function once hearing has been “restored.” If the extent of cross-modal re-organization is a limiting factor of post-treatment auditory outcomes, then aural rehabilitation or therapeutic techniques or treatments may help maximize treatment benefit for patients who may be struggling.

The reversal in cross-modal recruitment of auditory cortex by vision and reduction in pre-frontal and frontal cortex activity in the ARHL group after 6 months of hearing aid use suggests that restored audibility from well-fit hearing aids may promote more typical sensory functioning comparable to activity observed in the NH group at baseline. Future studies will aim to examine the role of pre-frontal and frontal cortex activity during sensory processing tasks in ARHL, as it relates to behavioral outcomes of auditory function and cognition. Future studies should also aim to understand how restored audibility with amplification alters top-down attentional or cognitive modulatory control of sensory function.

Finally, our results highlight the potential role of cognitive screening or evaluation in the clinical setting. Currently, only 25% of audiologists incorporate cognitive screening or other special tests into their clinical practice (Anderson et al., 2018). If treatment with hearing aids may provide neurocognitive benefit, then measuring cognitive abilities before and after intervention may provide an additional prognostic indicator or metric by which to evaluate post-treatment outcomes. For example, cognitive assessment tools may help audiologists make better recommendations regarding when a patient should receive intervention or help determine what kind of intervention or rehabilitation plan is ideal. Cognitive assessment may also be used to assess whether a selected intervention or rehabilitation method is providing sufficient benefit. The impact of hearing loss extends beyond the ear, impacting psychosocial function and cognitive function. Greater research resources should be devoted to understanding the larger impact of ARHL on health and wellness.

One of the most remarkable capabilities of the human brain is its capacity for change. As a profession, the field of audiology is beginning to unearth the widespread effects of hearing loss on structural and functional changes in the brain. Ultimately, our clinical interventions (e.g., hearing aids, cochlear implants) the neuroplastic ability for the brain to adapt to restored auditory input. With a more solid understanding of the mechanisms of neuroplasticity in ARHL, our profession may find new and innovative ways to leverage neuroplasticity in order to optimize treatment outcomes for our patients.

## Data Availability Statement

All datasets analyzed for this study are included in the manuscript.

## Ethics Statement

The studies involving human participants were reviewed and approved by the Institutional Review Board, University of Colorado Boulder. The patients/participants provided their written informed consent to participate in this study.

## Author Contributions

Both authors of this manuscript contributed to the design of the study, data analysis, interpretation, revision, and review of this manuscript.

## Conflict of Interest

The authors declare that the research was conducted in the absence of any commercial or financial relationships that could be construed as a potential conflict of interest.
